# Inference of Population History by Coupling Exploratory and Model-Driven Phylogeographic Analyses

**DOI:** 10.3390/ijms11041190

**Published:** 2010-03-24

**Authors:** Ryan C. Garrick, Adalgisa Caccone, Paul Sunnucks

**Affiliations:** 1 Department of Ecology & Evolutionary Biology, Yale University, New Haven, Connecticut 06520, USA; E-Mail: adalgisa.caccone@yale.edu (A.C.); 2 Australian Centre for Biodiversity, School of Biological Sciences, Monash University, Clayton, Victoria 3800, Australia; E-Mail: paul.sunnucks@sci.monash.edu.au (P.S.)

**Keywords:** cladistic analysis, landscape history, molecular markers, population structure, statistical phylogeography, temporal contrasts

## Abstract

Understanding the nature, timing and geographic context of historical events and population processes that shaped the spatial distribution of genetic diversity is critical for addressing questions relating to speciation, selection, and applied conservation management. Cladistic analysis of gene trees has been central to phylogeography, but when coupled with approaches that make use of different components of the information carried by DNA sequences and their frequencies, the strength and resolution of these inferences can be improved. However, assessing concordance of inferences drawn using different analytical methods or genetic datasets, and integrating their outcomes, can be challenging. Here we overview the strengths and limitations of different types of genetic data, analysis methods, and approaches to historical inference. We then turn our attention to the potentially synergistic interactions among widely-used and emerging phylogeographic analyses, and discuss some of the ways that spatial and temporal concordance among inferences can be assessed. We close this review with a brief summary and outlook on future research directions.

## 1. Introduction

Phylogeography focuses on understanding how population processes (e.g., gene flow) and historical events (e.g., vicariance or range expansion) influence the spatial distribution of biodiversity in extant species [1,2]. Although still relatively new, the discipline has made significant contributions to evolutionary theory. For example, empirical studies have provided historical frameworks for understanding the relative contribution of natural selection and genetic drift in driving speciation [3–6] or the strength of co-evolutionary associations among ecologically interacting species [7,8]. Comparative phylogeographic studies have also investigated whether multiple members of the same community responded to past landscape-level environmental changes in a concerted manner [9–13]. In addition to advancing evolutionary theory, phylogeography has direct applications in conservation biology. These include the identification of distinct intraspecific genetic units with unique evolutionary heritage that are otherwise overlooked by traditional morphotaxonomy, as well as the geographic centers of endemism that harbor them [14–19]. Molecular insights into organismal responses to past climate change are also relevant to predictive modeling of future impacts of global warming on species distributions [20–22]. A diverse set of empirical phylogeographic studies, including those with immediate conservation applications, have been comprehensively overviewed elsewhere [2]. Accordingly, the present review focuses on phylogeographic methods rather than applications.

Phylogeographic inferences are usually underpinned by DNA sequences assayed from the same locus for many individuals spanning the geographic range of a species. To identify the nature and magnitude of historical events that generated genetic structuring, analyses often make use of information embedded in phylogenetic relationships among DNA sequences (gene trees), the population frequencies of DNA sequence haplotypes, and/or the spatial locations from which they were sampled [2,23,24]. Previously, only the shape (*i.e.*, topology plus branch lengths) of gene trees reconstructed using molecular phylogenetic methods was used as the basis for inferring organismal history (e.g., vicariance [1], gene flow [25], population growth [26,27], effective population size [28]). However, statistical phylogeographic analyses have advanced over recent years [29–32], and researchers are now well-equipped to address questions that were previously intractable. This is due to the emergence of sophisticated model-driven approaches to estimating population genetic parameters and their confidence intervals, and this information can be used to generate more robust inferences [33–40]. The former gene tree-based approaches were qualitative and limited to general questions such as asking whether an extant species exhibits any spatial-genetic structure. Conversely, the newer model-driven methods are quantitative and permit landscape-specific questions (e.g., how is spatial-genetic structure distributed with respect to putative historical barriers?). Furthermore, complex population divergence scenarios can now be assessed by framing questions in a spatially- and temporally-explicit manner (e.g., are the empirical DNA sequence data consistent with a scenario of differentiation in separate refugia isolated during Last Glacial Maximum, followed by Holocene range expansion?). These developments have facilitated hypothesis-driven approaches to phylogeography.

The widespread adoption of model-driven coalescent methods represents a paradigm-shift in phylogeographic analyses, and this has been fueled by the recognition that stochasticity in the processes of lineage sorting and DNA sequence mutation can be large [29,41–43]. Stochasticity as a potential source of inference error can be partly accommodated *via* simulations under a particular divergence scenario or demographic model, and in this way the expected level of gene-to-gene variance is quantified [7,12,30,44–48]. Software for implementing these new approaches, together with publicly-available high-performance computational resources (e.g., Bioportal, University of Oslo, http://www.bioportal.uio.no; CBSU, Cornell University, http://cbsuapps.tc.cornell.edu), have also facilitated the transition to using model-driven coalescent methods for inferring population history.

The overall strength, accuracy and precision of any analytical method for reconstructing organismal history is likely to be determined by the nature of the questions at hand, and limitations of the available data [17,49]. Furthermore, the temporal and spatial scales under consideration, as well as species’ life history and dispersal biology, may be particularly important in dictating the appropriateness of an analysis method [50,51]. Coalescent methods can address a broad range of questions using a variety of molecular data, and their use is becoming standard practice in empirical phylogeographic studies. Nonetheless, these methods are computationally demanding and so it is necessary to make simplifying assumptions. This has put a premium on external information (e.g., non-genetic data) that can be used to narrow down hypotheses to a small set of biologically realistic *a priori* scenarios of population divergence, and/or to reduce the number of parameters that need to be estimated [52–54].

The degree to which model misspecification may generate spurious inferences when using some of the increasingly popular coalescent methods has not yet been well-studied. While evolutionary biologists await the outcome of rigorous performance-testing and sensitivity analyses using datasets simulated under a variety of historical scenarios, as well as empirical datasets for which the ‘truth’ is relatively well-known, it would be pragmatic to assume that no universally superior analytical approach exists. At the same time, there is an increasingly large battery of alternative methods that can be employed for analysis of population structure and demography [55,56]. To date, however, there have been few syntheses of how these alternative methods—each with different underlying models and assumptions—can be meaningfully integrated with one another. In the present paper, we review the strengths and weaknesses of different types of molecular data, as well as a suite of widely-used or emerging approaches to phylogeographic inference, and then highlight ways in which these different classes of analyses can complement each other. We also examine some of the challenges relating to assessing concordance among inferences drawn using different methods or datasets and integrating their outcomes, and suggest practical solutions.

## 2. Types of Genetic Data and Their Applications

**Figure 1.**
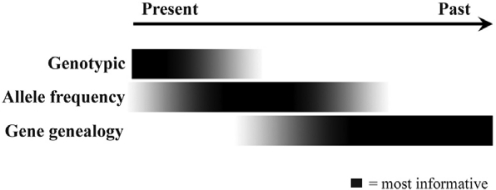
Three hierarchical levels of genetic information that can be obtained from diploid, co-dominant nuclear loci. Taken together, they cover a broad temporal spectrum, and the use of complementary analyses that focus on different ‘time slices’ of population history potentially allow these components to be separated.

Different classes of molecular markers provide insights into landscape-level barriers to gene flow and environmental changes that impacted connectivity among populations at contrasting temporal and spatial scales [2,49]. Whereas genotype and allele frequencies can change over few generations, DNA sequence mutations accumulate and spread throughout a population relatively slowly [57] (Figure 1). The combination of these data types not only provides a more complete understanding of how organisms responded to changes in the biogeographic landscape, but also potentially allows different components of species’ evolutionary history to be separated [13,17,49]. Below, we briefly summarize some characteristics and phylogeographic applications of each of the ‘three tiers’ of genetic information (*i.e.*, individual genotypes, population allele frequencies, and gene genealogies).

### 2.1. Individual Genotypes

The identity and configuration of the two alleles at a nuclear locus represents the genotype of a diploid individual. When genotypes are determined at several independent loci and considered jointly in an analysis, with sufficient density of sampling, these multi-locus genotypes can be very informative over fine spatial scales and short, generation-to-generation, ecological timescales (Figure 1). Over these spatial and temporal scales, individual-based analyses such as population assignment tests, relatedness and parentage analyses are commonly employed (e.g., suites of approaches in [58–60]). The information at this level of temporal process is associated with linkage disequilibrium (LD), *i.e.*, the correlation among alleles at different loci. The timescale reflected will depend on several factors including mating system, mobility, effective population size (*N_e_*) and physical linkage among loci, but LD among unlinked, neutral loci will decay at 50% per generation of random mating, declining to zero in less than 10 generations [61]. Multi-locus genotypes can be obtained by screening genetic variation at any set of unlinked nuclear loci that are effectively selectively neutral and exhibit Mendelian inheritance patterns, but interpretation is easiest and most powerful when both alleles in a heterozygous genotype can be observed (*i.e.*, when loci are co-dominant). Currently, microsatellites are the most widely-used type of molecular marker in this class, largely owing to their fast mutation rates and accessibility in non-model organisms. In out-crossing sexual species, the number and composition of distinct genetic clusters (or ‘populations’) can be determined directly from multi-locus genotype datasets. Some popular clustering methods are based on the null expectation of Hardy-Weinberg and linkage equilibrium within panmictic groups, and they implement algorithms that introduce structure into the dataset in response to deviations from these expectations [35,62]. In addition to population structure, multi-locus genotype datasets allow the identification of migrants, or admixed individuals (e.g., F_1_ and F_2_ hybrids) that are the result of breeding among members of different populations or species [63–66]. Different methods of assignment to genetic groups may be appropriate, depending on the structure of the sampling and data [50]. The same genotypic datasets can also provide novel insights into other aspects of population biology including sex-biased dispersal, mating systems, and detailed mechanisms such use of stored sperm [67]. Many of these insights would be impossible to obtain *via* direct observation, capture-mark-recapture techniques, or other nonmolecular methods.

It is important to note, however, that genotypic data are not without limitations. By itself, a single-locus genotype is usually not particularly informative about how an individual is related to others in a population sample. This necessitates the development and screening of a number of loci, often ≥5 microsatellite markers but many more for single nucleotide polymorphisms (SNPs), yet marker development in some organisms is notoriously difficult. Even after molecular markers have been developed and a modest number of population samples screened, it may then become apparent that some loci do not show classical Mendelian segregation, thereby undermining their utility. For example, loci may be affected by null alleles (e.g., those that do not amplify by polymerase chain reaction; PCR), or they may be physically linked to other assayed loci rendering them non-independent of each other. Some non-coding DNA regions such as microsatellites may even be physically linked to genes that are under strong selection, and so as a consequence of hitchhiking effects, the microsatellite may not behave as a neutral marker. Other complicating factors can include chromosomal location (e.g., sex-linked markers), and evolutionary history of the marker itself (e.g., screening of loci that are members of gene facilities can be challenging owing to co-amplification of paralogous alleles). Even well-behaved microsatellite loci can be difficult to score, and so a researcher usually needs to become very familiar with the morphology of allele peaks on an electropherogram, or banding patterns on a gel, in order to obtain accurate genotypic data. Finally, connectivity between datasets may become an issue if a locus has been screened over several years using slightly different PCR conditions and chemistry, or on different fragment analysis platforms.

### 2.2. Population Allele Frequencies

Whereas diploid genotypes of sexually reproducing individuals can be reconfigured every generation *via* intergenic recombination, population allele frequencies are usually less labile in the short-term [49] (Figure 1). For example, when *N_e_* is large, it can take a considerable amount of time for allele frequencies to diverge by genetic drift—even in the absence of gene flow [68]. Because population allele frequencies can be obtained directly from individual genotypes, the comparatively deeper temporal perspective from allele frequencies rather than individual-based genotypes can be extracted simply by employing the appropriate analyses. Indeed, numerous measures of among-population differentiation are based on allele frequency data (e.g., Weir and Cockerham’s [69] estimate of *F*_ST_; Cavalli-Sforza and Edwards’ [70] chord distance, *D*_C_; Nei’s [71] standard genetic distance, *D*_S_). Estimates of the effective number of migrants per generation (*N_m_*) can also be derived from population allele frequencies. In addition to allele frequency differences among populations, measures of within-population genetic diversity, such as allelic richness, can be informative about historical processes [48,72,73]. These can include demographic changes such as recent population contractions [74], and estimations derived from aspects of allele frequency, states and combinations [75]. Population Graphs [76,77] is a recent analytical development that draws on graph theory to estimate the minimum number of connections among sampled populations (*i.e.*, those linked by gene flow) that are necessary to explain the observed genetic covariance. This framework, which uses conditional genetic distance, opens the door to more sensitive tests of isolation-by-distance (c.f. pairwise *F*_ST_ or *D*_C_). Population Graphs can also provide novel insights into metapopulation structure, landscape-level barriers to dispersal, past vicariance, and the axes of range expansion [48,78,79]. Likewise, Amos and Manica [80] recently introduced a method that can be used to identify historical population centers.

Analyses based on frequencies of alleles at selectively neutral loci can interface very effectively with the individual-based approaches outlined in the previous Section. For example, comparing the estimates of dispersal and gene flow based on allele frequencies with those based on genotypic data can be informative about timescales over which changes in population structure occur [58]. Given sufficient density of sampling and genotypic power, it is becoming routine to estimate contemporary dispersal from direct genetic approaches rather than *via* indirect summary statistics such as *F*_ST_, although direct and indirect methods can be compared to good effect [81]. Frequency-based approaches remain useful where population sampling is not comprehensive, at broader geographic scales, deeper timescales, examining change over time, and for assessing functional genetic variation [82,83].

When new genetic variants arise in populations, they may increase in frequency and ultimately replace other variants. Thus there is an intrinsic overlap between frequency-based measures and those based on DNA sequence variation (Section 2.3). Untangling the temporal and spatial components is an issue of quantification. One useful approach to this is exemplified in the test of whether evolutionary information (DNA sequence variation, or microsatellite allele sizes—under the assumption that size similarity reflects shared ancestry of alleles) contains signal over and above that in frequencies only [84]. This approach can be used to scope whether population divergence has occurred on a timescale where genetic drift dominates to alter frequencies, or if limited gene flow has persisted over the timescale of evolution of new alleles at the relevant genetic markers [85].

Given that population allele frequencies are derived from individual genotypes, the same limitations mentioned in the previous Section also apply. However, some additional issues are also noteworthy. First, through the procedure of collapsing individual genotypes into population allele frequencies, there is a concomitant loss of information. For example, it is easy to imagine a situation where two groups of individuals have similar overall allele frequencies, yet the genotypic configurations of those groups are very different (e.g., one population containing a few long-distance immigrants that have not reproduced since arriving in their new location, *versus* another population with many admixed individuals carrying the genetic legacy of past introgression). Second, as a precursor to calculating allele frequencies, population boundaries must be clearly demarcated. Unfortunately, this enforces a dichotomous classification on all sampled individuals (*i.e.*, member or non-member or a particular group). In reality, however, population boundaries may be fuzzy and dynamic in space and time, and individuals may also be of mixed ancestry. In these cases, implementation of analyses that treat populations as the operational taxonomic unit may be challenging—even when biologically meaningful genetic clusters have been inferred from multi-locus genotypic data.

### 2.3. Gene Genealogies

DNA sequences represent the most common class of molecular data currently used in phylogeographic studies. Contiguous stretches of aligned homologous DNA characters assayed from an organellar or nuclear locus by direct sequencing are scored as haplotypes, and evolutionary relationships among haplotypes (*i.e.*, gene genealogies; Figure 1) can be estimated using maximum-parsimony, maximum-likelihood, Bayesian inference, or other molecular phylogenetic methods [86]. Alternatively, network approaches such as statistical parsimony [87] or median-joining networks [88] can be more appropriate for the typically shallow population-level sequence divergences because these methods allow for reticulation and the presence of extant ancestral sequences [89]. Furthermore, the root of an intraspecific network can be determined using predictions derived from coalescent theory [90,91], and this identification of ancestral polymorphisms in a contemporary gene pool contributes a temporal dimension to gene genealogies. Indeed, polarity of a network can be directly informative about historical events (e.g., relative or absolute timing of past vicariance, directionality of range expansions) or for distinguishing between contemporary *vs.* past gene flow [49]. Coalescent approaches based on gene genealogies have permitted explicit testing of the impacts of historical events on spatial patterns of intraspecific diversity, and in the following Sections, some exemplars of new and emerging coalescent methods are discussed in detail.

DNA sequence datasets are not free of drawbacks, and as with microsatellites, evidence for co-amplification of paralogues, or non-neutrality of loci, may emerge either during the screening process or from preliminary analyses. Similarly, intragenic recombination (usually affecting autosomal loci) may be difficult to detect with small sample sizes, and so considerable time and effort may have already been dedicated to screening a DNA sequence marker before problems are observed. If recombination is detected, the simplest course of action is identify putative cross-over points in the alignment, and then retain only the most information-rich Section of apparently non-recombining sequence for use in subsequent analyses. Another source of potential error associated with DNA sequence datasets is in the alignment of highly-variable non-coding regions. In addition to difficulties related to inferring homology of nucleotide substitutions when homoplasy is likely, insertion/deletion (indel) mutations can also be hard to align. Models of indel evolution are not yet available in phylogenetics software, and so this source of information is discarded even if it carries important historical signal. On a related issue, some phylogenetically-informative regions may be effectively inaccessible because they are interspersed with repetitive DNA that is very difficult to sequence through (e.g., long mono- or di-nucleotide repeats in chloroplast intergenic spacers and introns). However, perhaps the greatest technical challenge lies in generating multi-locus DNA sequence datasets, as this necessarily requires assaying nuclear loci. Even without the added complications contributed by allele size variation, the detection, scoring, and phase-determination of heterozygous sites from directly sequenced diploid PCR products (*i.e.*, recovering the true sequence of each of the two alleles at a locus) is considerably more labor-intensive compared to sequencing haploid organellar genes. In practice, even when using computational approaches to haplotype phase determination, at least some additional experimental verification needs to be conducted, and this requires physical isolation of alleles prior to sequencing (e.g., cloning, allele-specific PCR, or single-stranded conformation polymorphism). Highly variable loci tend to require more in-depth experimental verification owing to the large number of extant alleles and genotypes. In turn, this impacts that rate at which populations can be screened, and ultimately, the sample sizes obtained.

## 3. Classes of Phylogeographic Analysis Methods

In the present paper, we focus on general approaches to phylogeographic inference, rather than on details of the individual analysis methods that form the basis of these approaches. As a precursor to the next Section, here we briefly consider two broad classes of analysis methods: (1) ‘exploratory’ or minimally parameterized methods that are usually concerned with one simple component of evolutionary history, and (2) ‘model-driven’ methods that employ highly parameterized models to represent fully-defined population divergence scenarios (Table 1). We recognize that depending on their implementation, many of the analyses discussed below can span both categories. Indeed, all methods have implicit or explicit assumptions or underlying models of how molecular evolution and population divergence proceeds, but the dichotomy used here serves as a useful conceptual framework.

### 3.1. Exploratory Methods

In the absence of one or more *a priori* phylogeographic hypotheses, it is necessary to generate a working hypothesis *de novo*. In these cases, a basic understanding of the importance of putative landscape-level barriers to gene flow and impacts of past climatic or geological changes may be of primary interest. Exploratory methods can be used to assess evidence for past vicariance, range expansion or contraction and colonization, and to understand recurrent population processes. Using molecular data, exploratory methods facilitate the identification of key components of evolutionary history. For example, spatial-genetic structure can be examined in terms of the number, locations and members of distinct genetic clusters [35,62], the partitioning of diversity within and among populations [92], or genetic connectivity among populations [76,77]. This information can yield insights into the nature of long-term refugia or recolonization routes, and zones of secondary contact can be identified using admixture analyses. Population size changes are often associated with spatial expansion or contraction of a species’ range, and the outcomes of such events are amenable to investigation using both genotypic and genealogical data [26,27,74,93–97]. Finally, exploratory methods can provide information on intrinsic features of species biology (e.g., neighborhood size and dispersal [34,98], mating system and philopatry [59,60], or even reveal the existence of morphologically cryptic species [99]) that facilitates interpretation of results from other analyses.

Whereas exploratory methods are primarily concerned with a single component of evolutionary history, one method, Nested Clade Phylogeographic Analysis (NCPA [23,24]), considers evidence for several different components. At the time of its introduction, NCPA was unique in its ability to potentially separate multiple overlying processes and events (e.g., past vicariance, range expansion, restricted gene flow and dispersal), and also in its assessment of whether the empirical genetic dataset contained adequate sample sizes, genetic variation, and geographic coverage for meaningful historical inferences to be made. Interestingly, these two features are still unique to NCPA today. Evaluations of the method’s performance have largely focused on the single-locus implementation of NCPA, and while the implications of some alarming results from simulation studies remain a topic of debate, a renewed emphasis on the value of analyzing multiple independent loci [100] is warranted. Overall, the major strengths of exploratory methods lie in their broad applicability across diverse study systems, relatively few assumptions, and considerable scope for making unanticipated discoveries (Table 1). However, this flexibility comes at the cost of statistical discrimination among alternative explanations for a given phylogeographic pattern.

### 3.2. Model-driven Methods

Our ability to explore the full universe of phylogeographic scenarios for a species is limited—not only by the cost and time required to generate informative molecular datasets and the computational power needed to analyze them, but also by of the sensitivity of available summary statistics (e.g., for DNA sequences and their frequencies [45,101]). Model-driven methods attempt to overcome this problem by focusing computational resources and statistical power on examining a limited set of tractable scenarios that cover only a fraction of the parameter space associated with complex scenarios. In contrast to the ‘broad brushstrokes’ approach of exploratory methods in generating a phylogeographic scenario *a posteriori*, model-driven methods assess the support for well-defined *a priori* scenarios, and attempt to discriminate statistically among them [7,13,30,44–46,48,54,102]. The set of competing hypotheses may be derived from external information (e.g., dated fossils, known biogeographic events, or paleoclimatic reconstructions [10,52,53]), and the statistical tests are tailored towards examining aspects of species’ responses to past environmental change that are most relevant to the landscape system at hand. Alternatively, some model-driven methods assume a single *a priori* scenario, and then empirical data are fitted to it. In this case, estimates of the model’s parameter values and their confidence intervals are used to distinguish among historical scenarios that make contrasting predictions about these values. In addition to statistical discrimination among alternatives, an advantage of model-driven methods is that they explicitly account for some of the inherent noise in real genetic datasets that is a consequence of coalescent stochasticity (Table 1). However, even very simple models have many parameters, and so for purposes of computational tractability, most of these parameters must be treated as fixed—even if there is little information available to guide choice of these values. Accordingly, the potential error associated with model misspecification is a major concern when employing this class of phylogeographic analysis methods.

## 4. Approaches to Phylogeographic Inference

Some general approaches to historical inference rely more heavily on a single analysis method than do others, but most studies integrate different methods to some extent, with the aim of generating more robust inferences. In this Section, we overview widely-used approaches to phylogeographic inference and provide examples of empirical studies that have implemented them. One emerging approach—approximate Bayesian computation—is not covered here because its utility has largely been limited to tests of co-vicariance [101,103–106], and so there is little opportunity to assess strengths and weaknesses in the context of single-species studies (but see Templeton [107]). As in Section 3, the categories used here are necessarily broad or loosely defined and are intended to facilitate comparison.

### 4.1. Consensus Vote Approach

When several different analytical methods are available for examining the molecular signature of a particular historical event or process, a ‘consensus vote’ approach can be used to strengthen phylogeographic inferences. In the context of population growth, there are number of methods that exploit slightly different signal in the data. For example, changes in *N_e_* can be assessed from a sample of DNA sequences *via* the frequency distribution of haplotypes (e.g., Fu’s [94] *F_S_*), the frequency distribution of segregating sites (Tajima’s [93] *D*; Ramos-Onsins and Rozas’s [97] *R_2_*), or pair-wise nucleotide differences (mismatch analysis and the associated raggedness index [26,27]). In addition, coalescent genealogy samplers (e.g., fluctuate analysis [33] or Bayesian skyline plots [39]) can detect non-monotonic variation of *N_e_* over time. The latter methods also account for the stochastic branching of gene genealogies, and uncertainty around a maximum-likelihood or median point estimate is quantified *via* confidence intervals or posterior density distributions. Given this diversity of approaches, agreement between the results of different methods can be used as means of cross-validation [56]. This principle has been widely applied in the context of population growth [108–113]. Similarly, a considerable number of analyses with different underlying assumptions exist for detection of abrupt spatial-genetic discontinuities using multi-locus genotype or allele frequency data [35,62,76,114,115].

A practical limitation of the consensus vote approach is that an inferred historical event or process (or combination thereof) can be considered ‘strongly supported’ only when two or more alternative analytical methods that serve similar purposes are available. For example, the temporal assembly of simple components of evolutionary history (*i.e.*, past vicariance, range expansion, restricted gene flow and dispersal) is unique to NCPA [23,24], and so inferred sequence of events can be difficult to cross-validate using other methods (although cross-validation can be undertaken within NCPA over multiple genetic markers and co-distributed organisms). Even when several methods are available, it can be difficult to directly compare estimated parameters or summary statistics when their units or timescales differ (e.g., *N_e_* estimated over ecological *vs.* long-term evolutionary timescales [116]). Finally, there are some situations where a consensus vote approach could be positively misleading. For example, in the context of gene tree / species tree discordances, there are mechanisms by which the most likely gene tree topology does not match that of the true species tree [117].

### 4.2. Sequential Approach

Analytical methods that focus on different timescales can be used in combination with one another to permit inferences over a broad temporal spectrum, thereby making full use of the historical signal carried by molecular data [13,113,118]. When applied in a hierarchically nested manner (e.g., moving from ancient to more recent genetic patterns, or from broad to finer spatial scales), the insights of one method can be used to inform the focus, set-up, and interpretation of subsequent analyses. For example, it is common practice to start by assessing ancient subdivisions *via* phylogenetic analysis of DNA sequences, and if geographically localized clades are identified from the estimated gene tree, these are subsequently used as population units in analyses that focus on demography over intermediate timescales (e.g., coalescent estimators of migration rates or changes in *N_e_*). These same population units can then be re-examined using genotypic or allele frequency-based measures of population structure that are potentially informative about contemporary landscape-level barriers to gene flow (e.g., partitioning of variation [92]) or recurrent population processes (e.g., isolation-by-distance, differences in male- *vs.* female- or pollen- *vs.* seed-mediated gene flow).

The sequential approach to phylogeography is partly based on the principle of re-assessment and refinement of the current working hypothesis, as advocated by Buckley [119]. Each additional analysis contributes new information and clarifies interpretation, and there is likely to be at least some overlap in the historical signal that is captured by analyses that focus on different timescales [118]. However, unlike the consensus vote approach, the sequential approach typically lacks an assessment of how robust inferences made at each time period are to violations of assumptions. Furthermore, as with other descriptive approaches, errors can arise owing to stochasticity associated with gene coalescence and DNA substitution processes, and so the data may be subject to over-interpretation or confirmation bias [30–32,45]. Ultimately, alternative explanations for an observed pattern may be adequately considered.

### 4.3. Model Parameter Estimation

The availability of coalescent methods for estimating historical demographic parameters, and in some cases, the timing of past events such as population divergence or size changes, has increased dramatically over recent years [29,32]. Briefly, Kingman’s [120] coalescent uses a backwards-in-time approach to make predictions about neutral genetic variation present in a random population sample, and can be used to model aspects of that population’s history [121]. Recent attention has focused on behavior of the coalescent when some assumptions of the Wright-Fisher population model are violated—assumptions such as no geographic substructure [122], constant population size [33], and symmetrical migration among populations [36]. Most of the new and emerging coalescent methods use genealogy samplers to provide point-estimates of population genetic parameters, together with their confidence intervals [29,56]. The latter property, coupled with more realistic population models, contributes to the statistical rigor of historical reconstructions by explicitly modeling coalescent stochasticity [30–32,45]. The model parameter estimation approach to phylogeographic inference has been applied in both hypothesis-generating and hypothesis-testing contexts. Below, we focus on three widely-used coalescent analysis methods to highlight some of these diverse applications.

***fluctuate***—This software, now incorporated into the lamarc package [123], distinguishes past population growth (or decline) from size constancy over long-term evolutionary timescales. It jointly estimates maximum-likelihood values of the population mutation rate parameter, Θ (= 4*N_e_*μ for diploid autosomal genes, where μ is the per-site per-generation neutral mutation rate), and the exponential growth rate parameter, *g* (positive *g* = growth, negative *g* = decline). Simulations have shown that although values of Θ and *g* tend to be upwardly biased (*i.e.*, the software yields slightly positive *g* in the absence of growth), fluctuate performs modestly well when the true Θ-value is relatively large and DNA sequences from multiple unlinked loci are analyzed together [33].

In a comparative phylogeographic study, Lessa *et al.* [108] used fluctuate to investigate the causes of low genetic variation within populations of boreal North American mammals compared to high levels of variation seen in tropical Amazonian mammal species. Strong evidence for demographic growth based on mitochondrial DNA (mtDNA) sequence datasets was found only in the North American taxa, pointing towards a scenario of rapid northward range expansions following the retreat of Quaternary ice sheets in that region. In another study, this time centered in the Wet Tropics of north-eastern Australia, Hugall *et al.* [53] used fluctuate analysis of mtDNA from a low-mobility forest-restricted land snail to assess whether genetic data were consistent with predictions from paleoclimatic modeling of the species’ Quaternary habitat distributions. Forest patches that persisted as long-term stable refugia throughout the Last Glacial Maximum and then subsequently expanded during the Holocene were found to harbor snail populations that showed evidence for marked demographic growth, confirming the predictions of paleoclimatic projections. In the Lessa *et al.* [108] and Hugall *et al.* [53] studies, the signal of growth was very strong. However, given the aforementioned upward bias in estimated values of *g*, fluctuate could produce ambiguous results when expansion events were less pronounced. Carstens *et al.* [110] accommodated this source of potential error in their investigation into the phylogeography of a salamander species by simulating numerous sequence datasets under a model of population size constancy. For the purpose of realism, the simulated datasets were designed to match the characteristics of empirical mtDNA data (*i.e.*, estimated *N_e_* values, number of sampled individuals and DNA characters). These datasets were then analyzed using fluctuate to determine the null distribution of *g*-values relevant to the empirical salamander dataset. Ultimately, population size constancy was rejected, consistent with an *a priori* hypothesis of range expansion out of a mesic forest refuge in the Pacific Northwest of North America.

**Figure 2.**
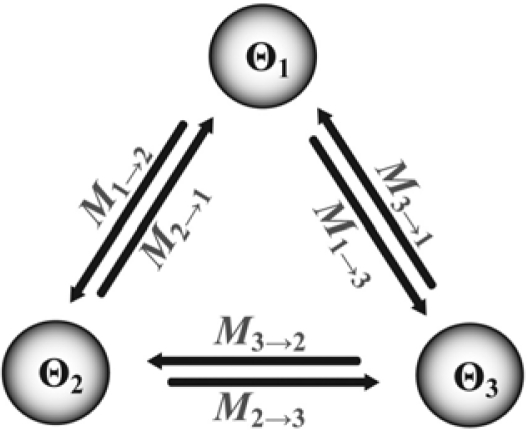
Example of a three-population migration matrix, showing parameters estimated by MIGRATE [36] when the full island model is implemented. There are three population mutation rate parameters (Θ = 4*N_e_*μ for diploid autosomal genes; one for each extant population) and six migration parameters (*M* = immigration rate divided by μ). Each population pair has two migration parameters to accommodate asymmetrical gene flow.

***migrate***—Unlike traditional estimates of migration rates drawn from molecular data, such as those derived from Weir and Cockerham’s [69] *F*_ST_, migrate can distinguish between gene flow into a recipient population, *versus* out of a source population [36]. The software estimates a migration matrix among *n* populations that have been exchanging genes for an indefinitely long period of time (where *n* is typically ≤ 10; Figure 2). In the simple case of a two-population model, migrate provides maximum-likelihood point-estimates and confidence intervals of the population mutation rate parameter for each extant population (*i.e.*, Θ_1_ and Θ_2_), and scaled migration rates *M*_1→2_ and *M*_2→1_ (where *M* is the number of effective immigrants per generation divided by μ, and directionality of gene flow from source to recipient is indicated by the arrow). migrate is particularly flexible in that it allows a user-specified migration matrix in which some *M*-values can be fixed at zero to produce 1- or 2-dimensional stepping stone models (c.f. the full island model). Also, some or all *M*- and Θ-values can be set as equal among population pairs (*i.e.*, symmetrical migration, *M*_1→2_ = *M*_2→1_; and/or same population mutation rate, Θ_1_ = Θ_2_). Likelihood-ratio tests can then assess whether a simplified matrix has a significantly worse likelihood score than the unconstrained (more complex) migration matrix, permitting statistical discrimination among phylogeographic scenarios that make different predictions about the parameter values. Using simulated datasets, Beerli [124] examined the effect of unsampled populations that contribute migrants into the sampled gene pool, and reported that although Θ-values were upwardly biased, *M*-values were relatively robust. However, an important limitation of migrate is the assumption that populations have been exchanging migrants for an indefinitely long period of time, at a constant rate. Potential sources of error included recently-diverged populations that have not yet reached equilibrium, episodic busts of migration, or human-mediated long-distance dispersal.

In a phylogeographic study of marbled murrelets from continental British Columbia and Alaska, and the western Aleutian Islands, Congdon *et al.* [125] estimated migration matrices using multiple unlinked DNA sequence loci. migrate analyses provided evidence for peripheral isolation of island populations on the basis of asymmetrical migration: island-to-mainland rates were consistently lower than in the reverse direction. Conversely, gene flow among mainland populations was relatively high, and based on the estimated *M*-values, this was sufficient to counteract the effects of drift among continental (but not island) populations. In another empirical application of migrate, Pfenninger and Posada [91] investigated postglacial recolonization routes of a land snail from southern European Pleistocene refugia. The authors used relative likelihood scores calculated for alternative migration models implemented in migrate to distinguish among a set of three phylogeographic scenarios. The analysis favored a source-sink island model, and the directionality of gene flow inferred by migrate was consistent with a scenario of northward expansion, as seen in other population-level studies in the region. In a comparative phylogeographic study, Garrick *et al.* [13] used migrate to examine congruence between two syntopic, rotting-log-dependent Collembola species (*i.e.*, soft-bodied Hexapods) from Tallaganda in southeastern Australia. Likelihood-ratio tests of constrained *versus* unconstrained migration matrices were used to assess the relative importance of two forest refuges as sources of recolonization of neighboring areas. Together with other analyses that focused on different timescales, migrate revealed that although the two species show similar spatial patterns of genetic variation, their demographic histories were idiosyncratic with respect to the locations of major refuges.

**Figure 3.**
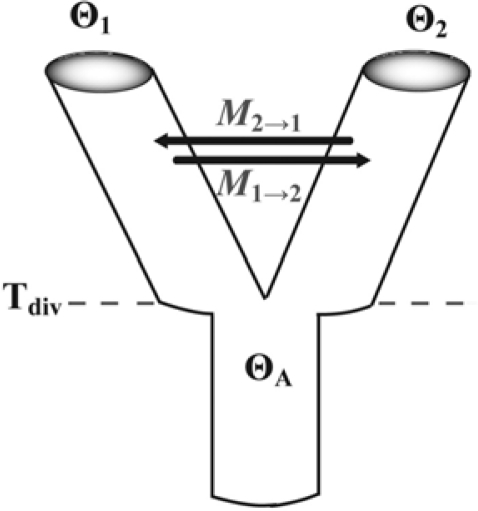
The six-parameter isolation-with-migration model implemented in IM and IMa [38,129]. There are three population mutation rate parameters (Θ), two migration parameters (*M*), and the time since population divergence (T_div_). In IM, an additional parameter—*s*, the proportion of the ancestral population that founds a descendant population—can be included to allow for population size changes (not shown).

***im***—Distinguishing between incomplete lineage sorting and gene flow has been a persistent challenge in phylogeography and related fields [113,126,127]. When genetic distances between populations are small, either recent divergence with zero gene flow (isolation model), or ancient divergence with low ongoing gene flow (migration model) can represent equally plausible explanations. To address this, Nielsen and Wakeley [128] developed mdiv, which has now been superseded by im [38] and ima [129]. The latter two programs implement a two-population isolation-with-migration model and are intended for the analysis of molecular data from a pair of extant sister populations that have diverged relatively recently (*i.e.*, gene trees are not reciprocally monophyletic), and which have not exchanged migrants with any other population except their immediate ancestor (Figure 3). From a practical perspective, there are two major differences between im and ima. The former allows for changing population sizes *via* an additional parameter (*s*, the proportion of the ancestral population that founded a descendant population), whereas the latter permits likelihood-ratio tests of nested models. Also of note, the genealogy sampler used in ima explores the parameter space more efficiently [129], which is important given the considerable computational demands of many coalescent analyses. In addition to the basic parameters estimated by migrate (*i.e.*, asymmetrical migration rates *M*_1→2_ and *M*_2→1_, and population mutation rate parameters Θ_1_ and Θ_2_), im and ima provide point-estimates and confidence intervals for the ancestral population mutation rate parameter (Θ_A_) and the time since divergence (T_div_). Unlike migrate, the isolation-with-migration model does not assume that populations are at mutation-drift equilibrium. The recent release of ima2 alleviates one of the major limitations of the method because it permits the analysis of more than two populations at a time. However, two simulation studies that assessed how robust parameter estimates generated by im are to violations of underlying assumptions reached rather disparate conclusions [130,131], suggesting that further work is needed to understand the impact of oversimplification of population processes.

Estimation of isolation-with-migration model parameters from empirical DNA sequence datasets now plays a central role in phylogeographic inference. Lee and Edwards [132] used im to investigate population divergence history of the red-backed fairy wren across a multi-taxon biogeographic break in northern Australia (the Carpentarian barrier). In addition to ongoing gene flow between populations, the authors found evidence for an unexpectedly recent initial splitting, which is at odds with divergence dates estimated for a co-distributed grass finch. Lee and Edwards [132] also showed that increasing the number of independent loci resulted in lower variance around parameter estimates, with the strongest improvement seen over the first 15 anonymous single copy nuclear loci. In another empirical application, Dolman and Moritz [133] used im model parameter values to compare demographic histories of rainforest skinks in the Australian Wet Tropics. In this landscape system, three mostly allopatric mtDNA lineages are separated by two well-characterized biogeographic breaks (Black Mountain Corridor and the Burdekin Gap). Two of these lineages are morphologically indistinguishable but show evidence for partial reproductive isolation at a narrow contact zone, whereas the third lineage is diagnosable on the basis of male throat coloration. Surprisingly, the two morphologically distinct lineages were found to have diverged most recently (*i.e.*, show a sister relationship). Furthermore, im indicated little or no apparent difference in *N_e_* across all lineages, which suggests that the relative contribution of drift is likely to have been equal across all lineages. As a result, this work highlighted the potential for different evolutionary outcomes to arise from the contrasting effects of extrinsic selection driving phenotypic stasis as opposed to geographically divergent intrinsic (e.g., sexual) selection. Muster *et al.* [134] used im in a phylogeographic investigation of a cold-adapted montane wolf spider. In this species, population connectivity was probably highest during periods of maximum ice sheet extent, and so this study represents a nice counter-point to the numerous studies that have uncovered evidence for divergence among populations isolated during Pleistocene glaciations. The principal question was whether relatively recent (*i.e.*, late Pleistocene / Holocene) or contemporary gene flow, *versus* longstanding genetic isolation coupled with incomplete lineage sorting, best explain the observed spatial-genetic patterns. Extensive simulations determined to what extent im parameter estimates could realistically discriminate among these and other competing scenarios, and the authors used this information to guide interpretation of results from im analysis of empirical data. Ultimately, scenarios that differed in the tempo of migration (*i.e.*, episodic burst *vs.* constant rate) and time since population splitting were not readily distinguishable.

Using three exemplar methods (*i.e.*, fluctuate, migrate and im), we have illustrated some diverse applications of the parameter estimation approach to phylogeographic inference, but their limitations warrant consideration. First, in most coalescent methods the basic underlying population model is forced on the data, yet it may nonetheless fit quite poorly [13,48,135]. Some common assumptions that will often be violated in real-world situations include panmixia within groups (no geographic structure or assortative mating), random sampling of individuals and genes (no family structure or age-cohort biases; no ascertainment bias affecting choice of loci), and the existence of crisp, clearly-demarcated population boundaries. The major effect of geographic structure within groups is to increase genetic variation (and estimated *N_e_*-values), and to extend coalescence times [136]. While impacts of other violations of coalescent assumptions are less clear, some insights may be gained from related genetic analysis methods. For example, in the context of genotypic clustering, family group structure represents a non-negligible source of error [137]. Similar conclusions have been reached for impacts of age-cohort and ascertainment biases on estimation of basic population genetic summary statistics [138,139]. Furthermore, the difficulty associated with defining population boundaries continues to attract considerable discussion, and there are reasons to believe that fuzzy boundaries are more biologically realistic than crisp boundaries [13,51,135,140]. However, because of the considerable computational burden associated with jointly estimating even a small number of parameter values, the model parameter estimation approach to phylogeographic inference necessitates using simplistic models that do not easily accommodate multiple overlying processes and events.

### 4.4. Simulations within Population Trees

**Figure 4.**
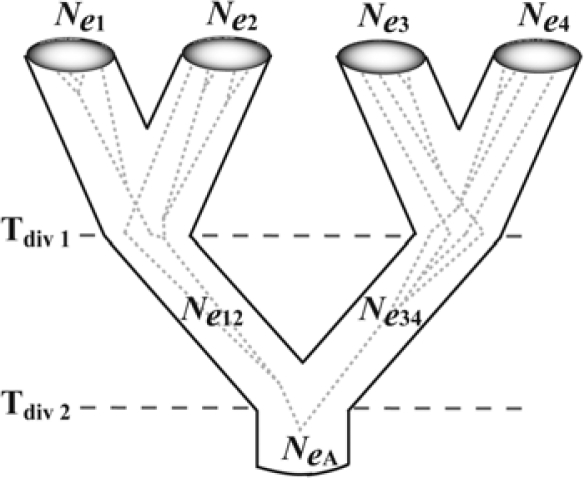
Hypothetical population tree containing a gene tree (dashed lines) that has been simulated *via* neutral coalescence. Even this relatively simple four-population model with zero post-divergence gene flow has many parameters that must be specified during construction of the population tree. Here, these include *N_e_*-values of four extant and three ancestral populations, two successive splitting times (T_div_), and tree topology. The population tree branch lengths are measured in organismal generations (scale not shown).

Hypotheses about population divergence history usually consist of information relating to the number, geographic locations, and timing of splitting events. Tree-like representations capture most of the key parameters needed to characterize a model of past vicariance, thereby providing a framework for phylogeographic inference using coalescent simulations [7,44,45,46,48,54,101,102]. On a population tree, tips are extant populations, nodes represent splitting events, branch lengths indicate divergence times, and branch widths reflect *N_e_* (Figure 4). Tree topology captures the temporal sequence of each divergence event, and the tree-like representation does not necessarily force a dichotomous structure on population divergences because polytomies are allowed. When two or more competing scenarios formulated on the basis of external information (e.g., dated fossils, known biogeographic events, or paleoclimatic reconstructions) can be represented as population trees, phylogeographic inference proceeds in the following way: (1) many gene trees are simulated within the constraints of each fully-defined population tree *via* neutral coalescence, (2) DNA sequence characters are evolved along the braches of the coalescent trees, (3) the simulated DNA sequence datasets are used to generate null distributions for the value of a chosen summary statistic under each scenario, and (4) the same summary statistic is calculated from the empirical DNA sequence dataset and then compared to the null distributions. Because each alternative scenario is treated as the null hypothesis when comparing observed (empirical) and expected (simulated) summary statistic values, the outcome of a test is to either ‘reject’ or ‘fail to reject’ the scenario at hand. The advantages of using simulations within population trees are that stochasticity associated with coalescent and DNA substitution processes are explicitly accommodated, divergence models can be tailored to reflect relevant components of the particular biogeographic landscape and species under study, and the underlying cause of spatial-genetic structure is directly linked to the hypothesis tests themselves.

In one of the first phylogeographic applications of coalescent simulations within population trees, Knowles [44] investigated whether Pleistocene glaciations promoted allopatric divergence among populations of a grasshopper species from the Rocky Mountains, USA. The ability to distinguish between a multiple refuge model and a single refuge model was assessed using simulated gene trees and associated values of Slatkin and Maddison’s [25] *s*—a summary statistic that measures the degree of discord between a population tree and a gene tree, assuming zero post-divergence migration. Despite considerable incomplete lineage sorting, the empirical mtDNA sequence data were consistent with a scenario of multiple refugia. DeChaine and Martin [7] used a similar approach to understand the degree of congruence with which an ecologically associated plant-insect species pair, also from the Rocky Mountains, had responded to Quaternary climatic oscillations. Alternative population divergence models based on geology and/or previous biogeographic studies in the region differed in the number of refuges and the partitioning of individuals among them. Simulations revealed that the host plant DNA sequence dataset was consistent with a two-refuge model only, whereas the pollinator data fitted both a two- and three-refuge model. Additional analyses supported that idea that although the plant and insect pair had responded similarly to past landscape-level environmental changes, some notable species-specific differences were evident too. Carstens and Richards [54] also used the coalescent simulation approach in a comparative phylogeographic context. However, in this case, paleoclimatic habitat distribution modeling was used to generate alternative hypotheses relating to ancient vicariance and recent dispersal of mesic forest biota in the Pacific Northwest, USA. This study focused on four distantly-related members of the same ecological community (*i.e.*, a frog, salamander, vole, and willow tree species), and used the same analytical framework for inferring population divergence history. The authors were able to demonstrate that, in some taxa, similar spatial-genetic patterns can emerge in the face of idiosyncratic ancestral distributions coupled with different demographic histories. This finding has far-reaching implications because it shows that if ‘biogeographic consensus’ is inferred for a given geographic region based only on spatial-genetic patterns, these conclusions may be erroneous. Similar findings have recently been reported in other taxa and landscape systems [13,141].

Despite the appeal of using coalescent simulations to test the fit of empirical data to alternative landscape-specific divergence scenarios, implementation of the approach can be quite challenging. The identification of simple yet biologically meaningful models that can be distinguished with the molecular data at hand is critical [30]. This requirement puts a premium on external information that can be used to narrow down the ‘universe’ of possible histories. Indeed, even simple population tree models have many fixed parameters, yet critical values such as contemporary and ancestral *N_e_*, or locus-specific per-generation mutation rates, are often not known [13]. Moreover, these methods cannot determine whether the conditions used in the models are correct, and more generally, any interpretive framework that requires the full historical scenario to be specified from the outset may fail if the true history is not included in the set of alternatives [107]. Accordingly, coalescent simulations within population trees are not particularly well-suited to making unanticipated discoveries.

### 4.5. Relative Ranking using Model Selection

In contrast to the simulations within population trees approach that is based on testing a series of null hypotheses one at a time, information-theoretic methods focus on evaluating the strength of empirical evidence in favor of each alternative *a priori* hypothesis included in a set of candidate models [142]. Rather than enforcing a ‘reject’/‘fail to reject’ dichotomy, information-theoretic approaches use model selection criteria to rank candidate models relative to one another. This ranking can then be scaled against the best model to provide an understanding about which hypotheses are only slightly worse, *versus* those that receive very little support from the empirical data [143]. Model selection methods have been widely used in ecology and evolution, and so a well-developed statistical framework exists [142,144,145]. In many cases, Akaike Information Criterion scores (AIC, or the related small sample criterion, AIC*_C_*) can be used as a measure of fit whereby complex models are penalized in proportion to the number of estimated parameters they contain. Through identifying the simplest model that best explains the data, the relative ranking approach can be used to understand the degree to which each of several historical events or processes (e.g., population divergence, size changes, gene flow) contribute to explaining observed spatial-genetic patterns.

Carstens *et al.* [146] examined the utility of model selection for phylogeographic inference in the context of ima’s isolation-with-migration model. Given an empirical multi-locus DNA sequence dataset from a mesic forest salamander, 16 different hypotheses (*i.e.*, alternative parameterizations of the im model) were ranked relative to the best-fit model *via* AIC, and then the overall probability of each one was quantified. Interestingly, rather than identifying a single best model, the authors found roughly equivalent support for two very different models—one that included non-zero post-divergence migration, and one that did not. Despite this ambiguity, all of the models that had a single value for the Θ parameter (*i.e.*, Θ_1_ = Θ_2_ = Θ_A_) fit the data very poorly, whereas all credible models allowed for low values of ancestral Θ compared to that of descendant populations (Θ_A_ < Θ_1_ and Θ_2_). This result was consistent with previous investigations of the species’ evolutionary history, which provided evidence for range expansion out of isolated refugia [12,54,110].

The coalescent simulations within population trees approach assumes that the particular scenario at hand is true and then calculates the probability of the data under this model, whereas the relative ranking approach calculates that probability of alternative models given the observed data [146]. One benefit of the relative ranking approach is that even when there are several competing models that cannot be rejected, the optimal model can still be identified. Also, model selection at least partly reduces the burden of specifying a set of fully-defined competing phylogeographic scenarios from the outset. As shown by Carstens *et al.* [146], nested or semi-nested models of increasing complexity can be assessed against one another. However, the approach is still fundamentally based on assessing a small number of *a priori* hypotheses, and so outcomes are heavily dependent on the quality of the candidate models that are included in the set [143]. Indeed, simply ranking unrealistic scenarios relative to one another, or rejecting an unrealistic scenario in favor of a more reasonable one, provides little insight into population history [30,144]. In the context of multi-species phylogeographic studies, another limitation is that information criterion scores cannot be compared across datasets [143].

## 5. Complementarity of Different Analysis Methods and Approaches to Phylogeographic Inference

Using a variety of genetic data types and analytical methods that exploit phylogeographic signal across broad temporal and spatial spectra generates opportunities for integrative approaches to historical inference [110]. For example, the outcomes of analyses focusing on genotypic and population allele frequency data can be used to validate or test key assumptions underpinning coalescent analyses of DNA sequences. The temporal contrasts made possible by differences in timescales over which the three hierarchical levels of genetic information are most useful also provide opportunities to assess whether recent demographic processes (e.g., migration, population size changes) reinforce rather than overwrite historical processes [6,13,48,64,109,113,147,150]. Ultimately, the complementarity of different datasets and analyses, coupled with an iterative approach to phylogeographic inference (*i.e.*, where data exploration precedes model-driven hypothesis-testing), should help to design an overall analytical framework focused on biologically-relevant scenarios [119]. Below we provide some specific examples of the potentially synergistic interactions among widely-used or emerging phylogeographic analyses.

### 5.1. Validation of Coalescent Assumptions

The subset of model-driven analyses introduced earlier (Sections 4.3 and 4.4) are representative of some common assumptions that are built into coalescent models of historical demography and population divergence. For example, the operational taxonomic units of these analyses are usually panmictic, unstructured populations or natural genetic clusters. These groups may be assumed to exchange no migrants with each other (e.g., fluctuate), to be at mutation-drift equilibrium and to have been exchanging migrants for an indefinitely long period of time (e.g., migrate), or to have exchanged genes only with a sister population and the immediate common ancestor (e.g., im). Other important assumptions can include constant population size over time, or that approximate *N_e_*-values are known for all extant and ancestral populations (e.g., coalescent simulations within population trees). Employing these model-driven coalescent methods necessarily incurs a cost—oversimplification of biological reality. However, the use of complementary analyses that are not subject to the same limitations can provide important insights into which underlying assumption(s) might be violated, and to what extent. Table 2 gives a non-exhaustive list of examples showing how the aforementioned model assumptions are amenable to validation or testing using freely-available analytical tools. Notably, the coupling of DNA sequence datasets with genotypic and population allele frequency information drawn from the same individuals provides considerable opportunities for cross-validation (Table 2). In addition to demographic assumptions, most coalescent methods also specify that DNA sequence loci are unlinked, selectively neutral, and recombination-free; a similarly diverse set of analyses also exists for testing these potential sources of model violation [125,133].

### 5.2. Focusing or Refining the Overall Analytical Framework

There are several advantages to formulating a set of competing phylogeographic scenarios *a priori*, and then attempting to distinguish among them in a hypothesis-testing framework [30]. We have provided some examples of how this can be achieved using null hypothesis-based approaches such as likelihood-ratio tests of constrained *versus* unconstrained estimated migration matrices [13] or nested isolation-with-migration divergence models [134], and also *via* coalescent simulations within population trees [7,44,48,54,102]. However, these approaches can be difficult to apply in study systems for which *a priori* hypotheses are not readily available owing to a lack of relevant external information [146,148]. For example, the generation of hypotheses about forces that drive divergence and speciation in forest fauna from a major biodiversity hotspot in south-western Australia has been complicated by a poor fossil record, long-term geological stasis, and an apparent absence of topographic barriers to gene flow [149]. Similarly, some studies are conducted over very fine spatial scales [13,112,113,150] and/or in habitats that are not particularly amenable to the insights that can be gained from paleoclimatic modeling [151,152]. Furthermore, while the use of model selection for ranking alternative phylogeographic scenarios is promising, so far it has been applied only in a study system that was already thoroughly explored and relatively well-understood [146]. Accordingly, in many empirical systems, exploratory analyses represent a valuable tool for generating working hypotheses that can be re-evaluated and refined using other methods. Identifying key components of population history *a posteriori* can help focus the overall analytical framework on biologically relevant phenomena.

As noted by Templeton [107], a major strength of NCPA is that the method can identify and assemble simple components of population history to build up a complex phylogeographic scenario, without the need for strong prior expectations. While this flexibility is appealing, we suggest that the method should be used as one of several approaches for reconstructing long-term population history [135]. Empirical studies have used NCPA as part of a battery of exploratory and model-driven analyses for assessing evidence for the signal of events such as range expansion or past vicariance [48,64,110,111,153]. However, this approach can involve little or no feedback between the results of one analysis, and the design of another. Alternatively, NCPA can guide the implementation of subsequent analyses by providing an estimate of the basic phylogeographic model. In turn, this can be refined by using model-driven coalescent methods for parameter estimation [13,91,112,113,154–156]. DeChaine and Martin [7] used NCPA inferences from their earlier phylogeographic studies [157,158], together with geological data and results from other genetic analyses, to characterize a set of models that they assessed using coalescent simulations within the context of a comparative study. In this way, the scenario inferred *via* NCPA can be treated as just one of several plausible historical scenarios, with model-driven hypotheses-testing approaches used to distinguish among them [91].

Evolutionary biologists want to avoid the undesirable situation where all of the scenarios under investigation are wrong (and not rejected). This necessitates performing at least some analyses that are capable of making unexpected discoveries. The examples outlined above illustrate the concept of complementarity: the strengths and weaknesses of NCPA have been debated elsewhere. When coupled with a basic understanding of species’ biology and landscape history, both the ‘consensus vote’ and ‘sequential’ approaches to phylogeographic inference should facilitate designing an analytical framework that identifies key historical events and processes that impacted genetic structuring of a species, or whole ecological communities (but see Section 7).

## 6. Assessing Concordance among Phylogeographic Inferences

Concordance of phylogeographic inferences can be considered at several levels of biological organization: among independent DNA sequence loci; across different types of genetic data (*i.e.*, genotypic, population allele frequencies and gene genealogies); or between co-distributed species that have evolved in the same landscape setting. Regardless of the level of biological organization, basic measures of concordance include the nature of the primary inference, degree of spatial co-occurrence, and temporal synchrony [37]. Vicariance and range expansion events have had considerable and lasting impacts on the spatial distribution of intraspecific biodiversity [2], and both are expected to leave genome-wide signatures [37,100]. Indeed, it is quite possible that different DNA sequence loci, genetic data types, or even species, mark the same historical event. However, for some aspects of vicariance and range expansion, quantitative methods for assessing spatial or temporal concordance are not yet well-developed. Here we explore some of the approaches that have been used to good effect in empirical phylogeographic applications.

### 6.1. Vicariance

Fragmentation of an ancestral population can result in two or more daughter lineages that now have allopatric distributions, separated by geographic areas in which the species is absent (e.g., newts [159], beetles [151], grasshoppers [44,47], isopods [152], butterflies [7,157], wolf spiders [134]). However, it is also relatively common for daughter lineages to exist in parapatry, with narrow zones of overlap formed on secondary contact (e.g., skinks [133,160], tree frogs [5,9], land snails [91], salamanders [161]). In either case, visual assessment of whether two or more gene trees estimated from independent DNA sequence loci exhibit the same branching order and geographically localized monophyletic clades (*i.e.*, temporal and spatial concordance) will usually be inadequate. Indeed, the error associated with phylogenetic estimation alone may account for any apparent incongruence. Parametric bootstrapping tests of monophyly [162] have been used in comparative phylogeographic applications to test for concerted responses to past landscape-level environmental changes [12,13,141,163]. Although not yet commonly used in the context of single species studies, this method should be equally well-suited to testing spatial congruence across independent gene trees drawn from different loci scored for the same individuals. Another versatile approach, boundary overlap analysis [164], is useful for testing whether locations of abrupt spatial-genetic discontinuities identified from two or more datasets show significant co-occurrence [13]. In contrast to parametric bootstrapping, boundary overlap analysis is concerned only with the spatial component of congruence, but could be used to directly compare outcomes from analyses of different types of genetic data (e.g., genotypic clustering *vs.* gene tree estimation). Temporal congruence of vicariance events inferred by two or more DNA sequence markers is amenable to testing *via* increasingly sophisticated molecular dating techniques—even when a strict clock-like substitution rate does not hold [40]. There is also some renewed interest in the utility of microsatellites for estimating dates of population splitting events [165], and this may provide a means of integrating inferences across different types of genetic data.

### 6.2. Range Expansion

Two different (albeit interrelated) components of a range expansion event are amenable to testing: evidence for population growth, and evidence for spatial expansion. There are several analytical methods available for addressing the former component using DNA sequences drawn from a single panmictic population (Section 4.1 and Table 2). Spatial congruence of inferences of population growth can be assessed qualitatively when the same operational taxonomic units (*i.e.*, natural genetic clusters) are used for the analysis of different DNA sequence loci and/or genetic data types. In the case of co-distributed species, however, the number of natural genetic clusters and their locations may show only partial or weak correspondence across taxa. To facilitate comparison in these cases, it may be necessary to sacrifice spatial resolution and assign each natural genetic cluster to a higher level geographic region based on proximity, after tests for population size changes have been performed. However, it will be important to ensure that these broad scale regions are still be biologically meaningful for the species under study (*i.e.*, defined by physiogeographic features or landscape history, c.f. political boundaries). Most of the methods for detecting the signal of growth from DNA sequences listed in Table 2 can also give some insight into the timescales on which growth occurred, and thus some indication of temporal congruence, provided that an estimate of the locus-specific substitution rate is available.

In contrast to growth, few methods explicitly assess evidence for spatial expansion (but see [23,24,148,166]). In terms of assessing congruence across loci or across different types of genetic data, establishing the polarity of a past range expansion (*i.e.*, source *vs.* advancing wave front) is likely to be particularly important. For example, although the expected mismatch distribution for a sample of DNA sequences can be derived under a continent-island model of spatial expansion [166], this approach does not provide insight into directionality of the expansion and so it is difficult to distinguish between scenarios where two or more loci mark the same event, as opposed to marking different events. However, coalescent methods for estimating asymmetrical migration rates provide a means of examining directionality [13,91,125,134]. Furthermore, comparison of genetic diversity statistics calculated from empirical data *versus* those simulated under alternative landscape-specific range expansion scenarios could provide a framework for assessing congruence across loci [167]. Even simple regression of within-population genetic diversity against geographic distance along the hypothesized axis of expansion can be very informative [48,72,73].

Despite the potential utility of molecular dating for examining temporal concordance among inferences of population growth or vicariance, which is a particularly promising endeavor when several external calibration points are available [152,159,168], caution is still warranted. Time-dependency of molecular rates can have non-negligible impacts on the accuracy of divergence dates estimated using intraspecific DNA sequence datasets [169,170], and the timescales that are most relevant to population-level studies occupy a critical region of the time-dependency curve. Another important consideration is that spatial patterns of genetic diversity resulting from range expansion can sometimes mimic that of past vicariance. Recently, a series of studies into a phenomenon dubbed ‘allele surfing’ have revealed that even a single uni-directional range expansion event can create complex spatial-genetic patterns that resemble segregation of clades that would usually be attributed to isolation in separate refuges [171]. The phenomenon is characterized by marked allele frequency changes that arise over short spatial and temporal scales, driven by strong genetic drift operating at the leading edge of a range expansion. That said, because this is a stochastic process, the spatial locations of apparent phylogeographic breaks generated by allele surfing should vary across loci and taxa. Even without a fully-developed analytical framework for quantitatively assessing temporal and spatial congruence, multi-locus studies should be less predisposed to spurious inferences [100].

## 7. Conclusions and Future Research Directions

A central theme of this review has been to highlight the complementarity of three hierarchical levels of genetic information (*i.e.*, genotypic, population allele frequency, and gene genealogies; Figure 1). Although the proportion of phylogeographic studies based exclusively on data from a single locus has decreased dramatically over the past 10 years [172], multi-locus datasets are still not necessarily being used to their full potential. Indeed, it could be argued that until inferences about microevolutionary processes operating over short, ecological timescales are routinely integrated with the longer-term historical perspective offered by gene genealogies, the original goal of phylogeography—to bridge the gap between population genetics and phylogenetics [1,2]—remains to be fully realized. New approaches to this endeavor will no doubt be motivated by the application of next-generation sequencing to rapid identification of perhaps hundreds of genotype-yielding, co-dominant nuclear SNP loci, together with phylogenetically-informative DNA sequence markers [173]. In the meantime, studies in which gametic phase of segregating alleles are determined for several independent nuclear DNA sequence loci (e.g., using laboratory-based or computational approaches [174,175]) could explore the possibility of using temporal contrasts between genotypic and genealogical information derived from the same locus to ground-truth empirical estimates of important population parameters. Genotyping of nuclear microsatellite or SNP loci from ancient DNA can also facilitate estimation of historical population parameters [65,66,176,177].

We have advocated a duality between what we loosely refer to as exploratory and model-driven analyses (Table 1). Over recent years, however, something of a false dichotomy has emerged in the phylogeographic community: methods that use heavily-parameterized coalescent models to test *a priori* null hypotheses have been implicitly (or explicitly) considered more valuable than exploratory methods. It is interesting to contrast this with the related field of molecular phylogenetics. Here, a *de novo* hypothesis about evolutionary relationships among species or higher taxa is generated using what is essentially a data exploration procedure (*i.e.*, tree searches with selected optimality criteria). The estimated phylogenetic tree is then used as a framework for making *a posteriori* inferences about biological phenomena as diverse as phenotypic trait evolution [178], the mode of spread of infectious disease [179], or the molecular mechanisms underlying occurrence of novel alleles found only in hybrid zones [180]. These exploratory approaches have provided many valuable evolutionary insights, many of which are unexpected.

Model-driven hypothesis-testing approaches are essential for discriminating statistically among alternative explanations for observed spatial-genetic patterns. However, to avoid a situation where the small set of alternative *a priori* scenarios under consideration do not capture the true history, the limited search of the phylogeographic scenario space needs to be conditioned on external information. Furthermore, given the potential for idiosyncratic process or events to exert strong impacts on genetic structuring of extant species, the basic phylogeographic model estimated using exploratory analyses of genetic data should at least be included in the set of alternative scenarios to be tested. This raises the issue of potential circularity, because data that generated a hypothesis are subsequently re-used to test it. However, this issue could be avoided by borrowing approaches from ecological modeling. For example, species-habitat relationship models are usually constructed by first identifying the basic parameters (or ecological predictor variables) with a training dataset, and these models are then re-evaluated using a test dataset [181]. An important point, from a practical perspective, is that models may be trained at a coarse spatial resolution but subsequently applied to previously unseen test data that were collected over finer scales [182]. Training datasets could also be applied in phylogeography, where a spatially representative subset of the sampled individuals (or assayed loci) are used to generate hypotheses, and then model-driven coalescent approaches subsequently re-assess these and other competing scenarios with a larger sample of individuals and/or loci.

In addition to the potential for exploratory and model-driven methods to be more fully integrated, simulated datasets can be used to determine false positive rates or discriminatory power—given the particular genetic dataset and scenario set at hand—for any analytical method. Several studies have demonstrated that simulations can be very effective in guiding the interpretation of results obtained from empirical DNA sequence datasets (e.g., fluctuate [110], im [134], NCPA [113,183]). Simulations have also been used to good effect for understanding the impact of violating one or more assumptions of the underlying model enforced by coalescent or other methods (e.g., im [130,131], migrate [124], species tree estimation [184]). Continued testing and refinement of new and emerging analytical methods is essential, but it would be useful to extend the criteria for validation beyond simulations under simple historical demographic scenarios, because these do not adequately capture the inherent noise in empirical datasets. Fortunately, some landscape systems represent excellent testing grounds for new methods. For example, linearly-arranged peninsulas or island chains can sometimes be treated as essentially one-dimensional, making them well-suited to assessing the fate of alleles during uni-directional spatial expansion or successive founding events. Indeed, there is a growing need for analytical methods that explicitly assess evidence for past spatial expansions, and infer their directionality, using a general framework that accommodates multiple data types [148]. Several other areas of analytical phylogeography warrant attention. For example, recent advances in landscape genetics [185] open the door to new approaches for quantifying the relative contribution of contemporary *versus* historical processes in shaping spatial-genetic structure [186], and in turn, this should generate insights into geographic scaling of microevolutionary processes [187]. Similarly, biophysical niche models that incorporate both physiological and spatial data can show greater correspondence with historical demographic or divergence scenarios estimated from genetic data (c.f. species distribution models that draw on presence/absence records only [154,188]). Accordingly, there is considerable scope for phylogeography to play an even more important role in bringing together not only population genetics and phylogenetics, but also physiology, ecology and geo-spatial sciences.

In closing, it is worthwhile to briefly reflect on the diverse applications for which these insights into organismal evolutionary history have been used, and to speculate on the impact that next-generation sequencing might have on the field in coming years. First, applications of phylogeography in conservation biology have included the identification of ‘evolutionarily significant units’ and ‘management units’ [14,17–19], delineation of geographic areas of high local endemism and landscape-specific recommendations for reserve design [15,16,189], and identifying individuals of high conservation value for inclusion in captive breeding programs [65,66]. Furthermore, an understanding or species’ responses to past climate change is directly relevant to predicting their responses to future changes [20–22]. Phylogeographic studies have also advanced our understanding of the geographic origins, dispersal routes, and modes of spread by invasive species [190], and provided information that is critical for effective control of disease vectors [147]. Some of the more recent applications model-driven phylogeographic analyses were reviewed by Knowles [193] and Hickerson *et al*. [32], and these authors all recognized that considerable advances had been made over a relatively short time. Taken together, it is clear that phylogeography will continue to be an important discipline-bridging field that contributes to evolutionary theory and applied conservation biology.

With the emerging promise of next-generation sequencing, perhaps the most immediate benefits to phylogeography will be the rapid development of nuclear markers for non-model species [194,195]. Importantly, next-generation sequencing datasets can be used for several purposes. For example, the same contig assemblies that are used for microsatellite and SNP discovery can also be mined for phylogenetically-informative introns. This represents a considerable advance over the exon-primed intron-crossing PCR approach to marker development, not only for taxa that have so far shown little nuclear DNA sequence variation at well-characterized introns [196], but also for groups that are underrepresented in genomic databases and for which sets conserved PCR primers are unavailable. Nonetheless, work is needed to empirically determine error rates and to establish genotyping standards (e.g., minimum depth of coverage), and also to come to terms with how ascertainment bias can be mitigated [139,165]. A number of other technical and analytical challenges need to be overcome. For the purpose of generating suites of neutral population-genetic markers in non-model species, it would be desirable to develop a general-purpose pipeline that does not require prior information on the size of the organism’s genome, or depend on the existence of a fully- or partially-sequenced genome from a closely related species. Reduced representation libraries [197] provide a means of reducing the size and complexity of the genome prior to sequencing, and the increasing average read lengths of most platforms will greatly facilitate *de novo* assembly and alignment of contigs. Ultimately, the traditional separation between marker development and population screening may become obsolete if read lengths and coverage are significantly improved over coming years, to the extent that high throughput genotyping of SNPs, microsatellites and gene genealogy-yielding DNA sequence makers can be conducted without first enriching for these genomic regions using PCR, gene capture on microarray chips, or other time consuming pre-sequencing steps. While the next-generation sequencing technology is bound to continue to improve rapidly and running costs should also decrease, its application to phylogeography will require flexible bioinformatics software tailored towards evolutionary biologists who work with non-model organisms, as well as new phylogeographic analyses that can accommodate unprecedented amounts of data. The field is developing very rapidly, and it has the potential to integrate the power of cutting-edge genomics technology with long-standing Earth science disciplines.

## Figures and Tables

**Table 1.** Characteristics of two major classes of phylogeographic analysis. Although ‘exploratory’ and ‘model-driven’ analyses are not mutually exclusive, the dichotomy can serve as a conceptual framework.

**Characteristic**	**Class of analysis**	
	**Exploratory**	**Model-driven**
*Level of Parameterization*	Low	High
*Reliance on a priori assumptions*	Low	High
*Multiple events or population processesa*	No	Yes
*Coverage of total ‘scenario space’*	Broad	Narrow
*Permits unexpected discoveries*	Yes	Limited
*Accommodates stochasticity*	Limited	Yes
*Statistical discrimination among alternatives*	Limited	Yes
*Framework for comparisons across species*	Qualitative	Quantitative

a NCPA is considered ‘exploratory’ here and is unique in its ability to separate multiple temporally overlying events and processes. Conversely, several model-driven methods explicitly consider temporally sequential events or processes (e.g., IM, simulations within population trees; Sections 4.3 and 4.4).

**Table 2.** Complementarity of phylogeographic analyses. Assumptions enforced by some coalescent methods can be validated or tested using other methods. This table is intended only as an example of some of the analytical methods that can be used to complement one another. Additional analytical resources are overviewed by Excoffier and Heckel [55], and Kuhner [56]. All of the software listed in this table is freely-available, and the associated references and websites are given in Supplementary Material (Table S1).

**Assumption**		**Validation using genotypic or allele frequency data**	**Software**	**Validation using gene genealogies**	**Software**
**Finding groups**	Natural clusters exist	Genotypic clustering a Allelic clustering a Spatial-genetic discontinuities a Population Graphs a	STRUCTURE, GENELAND SAMOVA BARRIER GENETICSTUDIO	Spatially cohesive clades Allelic clustering a Spatial-genetic discontinuities a	GARLI, MR BAYES, BEAST SAMOVA BARRIER
**Within groups**	Random mating	Hardy-Weinberg and Linkage Equilibrium	ARLEQUIN, GENEPOP, FSTAT		
	No geographic structure	Spatial autocorrelation a Isolation-by distance a and spatial-genetic gradients a	GENALEX IBD, ARLEQUIN, GENEPOP, FSTAT, GENETICSTUDIO	NCPA a Isolation-by distance a and spatial-genetic gradients a	TCS and GEODIS IBD, ARLEQUIN, GENEPOP, FSTAT, GENETICSTUDIO
	No family structure	Relatedness analysis a	KINGROUP		
	Constant size	Recent growth or decline	BOTTLENECK, MSVAR	*F*_s_, *R*_2_, *D* or mismatch analysis Growth rate (*g*) estimation Bayesian skyline plots	DNASP FLUCTUATE BEAST
	*N*_e_ is known	Θ estimation b *N*_e_ estimation	IM, MIGRATE ONESAMP, LDNE	Θ estimation b	IM, MIGRATE, FLUCTUATE
**Among groups**	No migration	Isolation-with-migration analysis Bi-directional migration rates Symmetrical migration rates Genotypic clustering a Assignment tests	IM MIGRATE BAYESASS STRUCTURE, GENELAND GENECLASS	Isolation-with-migration analysis Bi-directional migration rates Reciprocally monophyletic clades	IM MIGRATE GARLI, MR BAYES, BEAST
	Sister relationship	Distance-based clustering	PHYLIP	Species tree estimation c	AUGIST, BEST, STEM
	Old divergences	Microsatellite dating: (δµ)^2^ or *T*_D_^d^		Relaxed-clock molecular dating	BEAST, R8S

a Analysis requires geo-referenced genetic data;

b Converting Θ to *N_e_* requires an estimate of per-locus mutation rate, and organismal generation time;

c Requires multiple unlinked, recombination-free, selectively neutral DNA sequence loci;

d Refer to Goldstein *et al*. [191] and Zhivotovsky [192], respectively.
